# Antifungal *Streptomyces* spp. Associated with the Infructescences of *Protea* spp. in South Africa

**DOI:** 10.3389/fmicb.2016.01657

**Published:** 2016-11-02

**Authors:** Zander R. Human, Kyuho Moon, Munhyung Bae, Z. Wilhelm de Beer, Sangwon Cha, Michael J. Wingfield, Bernard Slippers, Dong-Chan Oh, Stephanus N. Venter

**Affiliations:** ^1^Department of Microbiology and Plant Pathology, Forestry and Agriculture Biotechnology Institute, University of PretoriaPretoria, South Africa; ^2^Natural Products Research Institute, College of Pharmacy, Seoul National UniversitySeoul, Republic of Korea; ^3^Department of Chemistry, Hankuk University of Foreign StudiesYongin, Republic of Korea; ^4^Department of Genetics, Forestry and Agriculture Biotechnology Institute, University of PretoriaPretoria, South Africa

**Keywords:** *Sporothrix*, *Candida albicans*, antifungal, *Protea*, infructescence, fungichromin, actiphenol, *Streptomyces*

## Abstract

Common saprophytic fungi are seldom present in *Protea* infructescences, which is strange given the abundance of mainly dead plant tissue in this moist protected environment. We hypothesized that the absence of common saprophytic fungi in *Protea* infructescences could be due to a special symbiosis where the presence of microbes producing antifungal compounds protect the infructescence. Using a culture based survey, employing selective media and *in vitro* antifungal assays, we isolated antibiotic producing actinomycetes from infructescences of *Protea repens* and *P*. *neriifolia* from two geographically separated areas. Isolates were grouped into three different morphological groups and appeared to be common in the *Protea* spp. examined in this study. The three groups were supported in 16S rRNA and multi-locus gene trees and were identified as potentially novel *Streptomyces* spp. All of the groups had antifungal activity *in vitro*. *Streptomyces* sp. Group 1 had inhibitory activity against all tested fungi and the active compound produced by this species was identified as fungichromin. *Streptomyces* spp. Groups 2 and 3 had lower inhibition against all tested fungi, while Group 3 showed limited inhibition against *Candida albicans* and *Sporothrix* isolates. The active compound for Group 2 was also identified as fungichromin even though its production level was much lower than Group 1. The antifungal activity of Group 3 was linked to actiphenol. The observed antifungal activity of the isolated actinomycetes could contribute to protection of the plant material against common saprophytic fungi, as fungichromin was also detected in extracts of the infructescence. The results of this study suggest that the antifungal *Streptomyces* spp. could play an important role in defining the microbial population associated with *Protea* infructescences.

## Introduction

Species of the genus *Protea* (Proteaceae) are shrubs and small trees distributed throughout sub-Saharan Africa (Rourke, 1980). A total of 114 species are known of which the greatest diversity occurs in the Cape Floristic Region (Rourke, 1980). The flowers of *Protea* spp. are arranged into inflorescences (Rourke, 1980) and the mature seeds are stored for long periods of time in these structures before they are released when environmental conditions are suitable for seed germination (Bond, 1985). Mature, closed inflorescences containing seeds are referred to as infructescences. The infructescences of many species remain closed until the time of dispersal and consist of abundant dead plant tissue in a moist, warm, and often insect-colonized environment.

A remarkable feature of the closed infructescences of *Protea* spp. is that very few fungi are found colonizing them at any given time, despite the fact that they provide the perfect conditions in which saprotrophic fungi would typically thrive (Marais and Wingfield, 1994; Zhou and Hyde, 2001; Roets et al., 2013). One would expect to find common saprophytes such as species of *Trichoderma, Penicillium* and the many other fungi frequently encountered under similar conditions in nature, but this is not the case. A number of studies have shown that before onset of senescence this enclosure is mainly occupied by a specialized group of ascomycete fungi, often referred to as the ophiostomatoid fungi, belonging to either the Ophiostomatales or Microascales (Marais and Wingfield, 1994; Marais et al., 1998; Wingfield et al., 1999; Roets et al., 2008, 2010, 2013). The ophiostomatoid associates of *Protea* infructescences from Southern Africa mainly reside in the genera *Sporothrix* and *Knoxdaviesia* (= *Gondwanamyces*) (Marais and Wingfield, 1994; Marais et al., 1998; Wingfield et al., 1999; Roets et al., 2008, 2010, 2013; de Beer et al., 2013a,b, 2016).

Our hypothesis to explain the absence of saprophytes in the infructescences is that antibiotic producing bacteria may suppress their growth in these environments. The aim of this study was to consider whether antibiotic producing bacteria occur in the infructescences of *Protea repens* and *P. neriifolia*. The antifungal activity of such isolates against common saprophytes and the ophiostomatoid associates that are typically present were also determined.

## Materials and methods

### Isolation of bacterial strains

**Figure 1:**
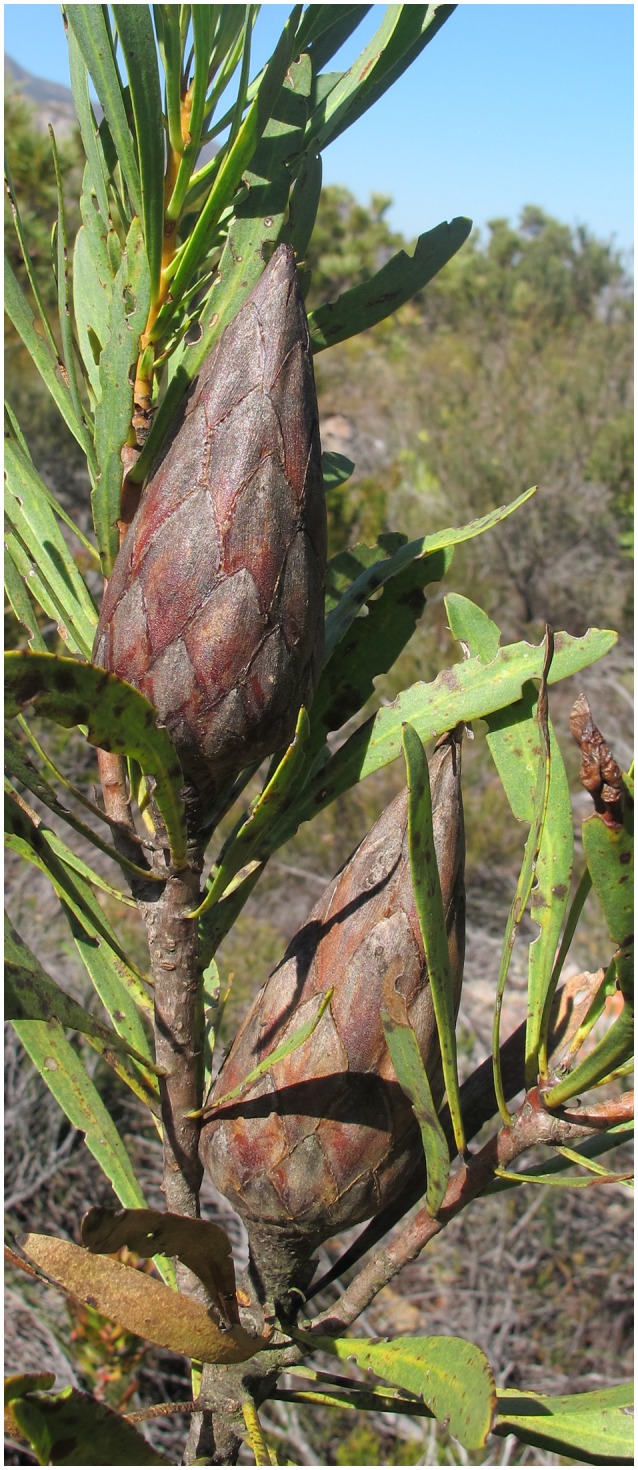
**A *Protea repens* branch with infructescences**.

*P. repens* infructescences (Figure 1) were collected at Pringle Bay and at the J.S. Marais Botanical gardens near Stellenbosch, and *P*. *neriifolia* infructescences from Pringle Bay and Franschhoek, in the Western Cape Province of South Africa. The infructescences were aseptically opened and parts from the individual flowers, outer involucral bracts, inner involucral bracts, and involucral receptacle were removed. Tissue samples from the different floral parts were suspended in a solution of 6% yeast extract and 0.05% SDS (Sodium Dodecyl Sulphate) and incubated at 40°C for 20 min (Hayakawa and Nonomura, 1989) followed by dilution to 10^−6^ to remove the antimicrobial detergent effect of SDS (Hayakawa and Nonomura, 1989). These suspensions were then plated onto three types of artificial media: YEME as described by Cafaro and Currie (2005), glycerol-asparagine agar (Pridham and Lyons, 1961) and starch casein agar (Kuster and Williams, 1964).

Plates were incubated at 28°C for approximately 14 days, whilst being continuously monitored. Colonies provisionally identified as actinomycetes based on morphology were obtained in pure culture by repeated isolation on YEME (Cafaro and Currie, 2005). Isolates were then grouped based on similar colony morphology. Representatives were selected from each group for further study. Care was taken to include isolates from each host species and location sampled.

### DNA extraction and sequencing

Genomic DNA was isolated by adding mycelium from each single pure colony into Prepman Ultra™ solution (Applied Biosystems). For identification of isolates, the 16S ribosomal RNA gene region was selected for sequencing. For further phylogenetic placement of isolates, the *trpB, rpoB*, and *gyrB* gene regions were amplified. The 16S rRNA gene region was amplified using primers pA and pH designed by Edwards et al. (1989). The methods from Guo et al. (2008) were used for the *trpB* and *rpoB* PCR reactions and those from Rong et al. (2009) for the *gyrB* gene. All reactions were performed using SuperTherm *taq* polymerase (Southern Cross Biotechnology, Cape Town, South Africa) on an Applied Biosystems Veriti Thermal Cyler. The 16S rRNA (Edwards et al., 1989), *gyrB* (Rong et al., 2009), *rpoB* and *trpB* (Guo et al., 2008) amplicons were sequenced using the methods previously described. Purified PCR products were sequenced using the ABI BigDye system (Applied Biosystems) and purified using sodium acetate precipitation. Sequences were analyzed on an ABI 3130 sequence analyzer (Applied Biosystems).

### Phylogenetic analysis

Isolates obtained were initially identified by comparing their 16S rRNA sequences to all type strain sequences available in the RDP database using the Seqmatch program hosted by the Ribosomal Database Project II (RDP-II) (Maidak et al., 2001). The closest matching sequences of validly described species were downloaded and included in a single data set with our sequences. *Mycobacterium tuberculosis* was used as an outgroup.

The sequences for the 16S rRNA, *gyrB, rpoB*, and *trpB* (Supplementary Table S1) were concatenated along with those from selected reference strains using SequenceMatrix software (Vaidya et al., 2010). Sequence ends were trimmed using Bioedit (Hall, 1999) and the sequences aligned using MAFFT (Katoh et al., 2005). The best-fitting nucleotide substitution model was determined using JModeltest version 0.1 (Posada, 2008) and selected using the Akaike Information Criteria (Posada and Buckley, 2004). A maximum-likelihood phylogeny was produced with PhyML version 3.0 (Guindon et al., 2009) using the TIM3 nucleotide substitution model for 16S rRNA and the TVM+I+G nucleotide substitution model for the concatenated data set (Guindon et al., 2009; Darriba et al., 2012). Tree confidence was measured using a bootstrap search with 1000 replicates (Felsenstein, 1985). The tree was visualized using Mega 4 (Tamura et al., 2007).

### *In vitro* antifungal assays of *Streptomyces* spp. strains from *Protea* infructescences

Antifungal bioassays were performed using the method described by Cafaro and Currie (2005). Bacterial isolates used in bioassays were randomly selected, with at least two isolates chosen from each group (Table 1). The fungal isolates selected were representative of fungi occurring in infructescences of *Protea* spp. and ubiquitous saprophytic fungi. *Knoxdaviesia proteae* and *Sporothrix splendens* are the most common fungi in the infructescences of *P. repens*, and *S*. *phasma* is common in *P*. *neriifolia* (Roets et al., 2009). *Fusarium* and *Penicillium* spp. are ubiquitous saprophytic fungi and have occasionally been reported from the infructescences of *Protea* spp. (Lee et al., 2005; Visagie et al., 2014). *Candida albicans* is sensitive to many antifungal compounds and is a common isolate used in antifungal assays which could serve as a control for antifungal activities observed. All fungi were retrieved from the CMW culture collections (Forestry and Agriculture Biotechnology Institute, University of Pretoria, South Africa; Table 2), except for *C. albicans* (retrieved from the ATCC) and the *Penicillium* sp., which was isolated from a *P*. *repens* infructescence (data not shown). The clear zones between fungal growth and putative antibiotic producing bacterial isolate were measured. This is referred to as the zone of inhibition and is used as an indication of the potency of antibiotic and the level of susceptibility of the test organism (Cafaro and Currie, 2005).

**Table 1:** **Isolates of *Streptomyces* obtained during the present study, their respective sources, and groupings based on culture morphology and 16S rRNA gene sequences and potential antifungal compounds**.

**Group**	**Isolate**	**Host**	**Location**	**Genbank nr**.	**Active compound**
1	PrRe2I4	*P. repens*	Pringle Bay	KX685570	Not tested
1	PrRe3I9	*P. repens*	J.S. Marais Gardens, Stellenbosch	KX685572	Not tested
1	PrRe2I22	*P. repens*	Pringle Bay	KX685571	Fungichromin
1	PrRe3I7	*P. repens*	J.S. Marais Gardens, Stellenbosch	KX685568	Not tested
1	PrNe0I20	*P. neriifolia*	Franschhoek	KX685569	Fungichromin
1	PrRe3I4	*P. repens*	J.S. Marais Gardens, Stellenbosch	KX685567	Fungichromin
2	PrNe0I9	*P. neriifolia*	Franschhoek	KX685576	Not tested
2	PrNe0I10	*P. neriifolia*	Franschhoek	KX685574	Not tested
2	PrRe2I24	*P. repens*	Pringle Bay	KX685575	Not tested
2	PrNe1I9	*P. neriifolia*	Pringle Bay	KX685577	Not tested
2	PrRe1I17	*P. repens*	J.S. Marais Gardens, Stellenbosch	KX685573	Unknown
3	PrRe3I6	*P. repens*	J.S. Marais Gardens, Stellenbosch	KX685579	Actiphenol
3	PrNe2I2	*P. neriifolia*	Pringle Bay	KX685582	Not tested
3	PrRe3I13	*P. repens*	J.S. Marais Gardens, Stellenbosch	KX685580	Not tested
3	PrRe4I4	*P. repens*	Pringle Bay	KX685581	Not tested
3	PrRe1I9	*P. repens*	J.S. Marais Gardens, Stellenbosch	KX685585	Not tested
3	PrRe3I1	*P. repens*	J.S. Marais Gardens, Stellenbosch	KX685584	Not tested
3	PrRe4I7	*P. repens*	Pringle Bay	KX685583	Not tested
3	PrRe3I5	*P. repens*	J.S. Marais Gardens, Stellenbosch	KX685578	Not tested

**Table 2:** **Zone of inhibition recorded in bioassay pairings**.

		**Group →**	**1**	**1**	**1**	**2**	**2**	**3**	**3**
	**Species ↓**	**Isolates ↓ →**	**PrRe2I22**	**PrRe3I4**	**PrNe0I20**	**PrRe1I17**	**PrNe1I9**	**PrRe3I6**	**PrRe4I4**
**FUNGAL ISOLATES**	*Fusarium* sp.	CMW19975	18.70	16.63	16.08	7.58	10.92	19.42	17.48
	*Penicillium* sp.		17.12	16.43	18.53	8.18	13.18	13.88	18.45
	*C. albicans*	ATCC 10231	22.30	16.85	13.93	13.43	11.87	10.38	7.38
	*S. splendens*	CMW23050	19.62	21.50	21.23	16.14	15.80	18.65	15.63
	*S. splendens*	CMW20680	20.43	20.97	22.57	17.32	15.45	14.25	12.03
	*S. phasma*	CMW20686	22.10	19.13	19.78	15.30	12.03	15.13	12.25
	*S. phasma*	CMW20676	24.83	19.80	21.75	14.72	21.43	11.18	11.20
	*K. proteae*	CMW40883	22.30	20.70	21.50	16.67	15.32	18.10	15.63
	*K. proteae*	CMW40884	21.12	21.85	20.85	14.36	14.83	18.34	15.50

*Values are averages of at least three replicates in mm*.

### Profiling secondary metabolite production of *Streptomyces* spp.

All the *Streptomyces* strains were cultivated in 200 mL YEME (4 g yeast extract, 10 g malt extract, and 4 g glucose in 1 L distilled water) medium in 500 mL Erlenmeyer flasks. The profiles of secondary metabolites of the bacterial strains were analyzed by LC/MS every 2 days. When the cultures were monitored, 10 mL aliquot of each culture was extracted with 10 mL of ethyl acetate (EtOAc). The extracts were concentrated *in vacuo* and resuspended in 1 mL of methanol (MeOH). After filtering the methanol-soluble extracts, 10 μL from each concentrated extract in MeOH was injected to LC/MS (Agilent 1200 Series HPLC coupled with Agilent 6130 single quad mass spectrometer). Gradient solvent conditions were consistently used (0~20 min: 10~100% CH_3_CN in H_2_O, 20~25 min: 100% CH_3_CN/flow rate: 0.7 mL/min, 0.1% formic acid in CH_3_CN and H_2_O). A diode array UV detector scanned from 200 to 700 nm. The mass spectrometric analyses were performed on both positive and negative ion modes. The acquired LC/MS profiles were analyzed with Agilent Chemstation 4.3 software.

### Identification of major secondary metabolites from *Streptomyces* spp. strains

The UV spectra of the major common secondary metabolites detected in the LC/MS chemical profiling were compared with the house-built UV spectral library interlinked with Agilent Chemstation 4.3 software. For further analysis, the major secondary metabolite of *Streptomyces* Group 1 was produced by large-scale (18 L) cultivation of a representative strain (PrRe2I22). The whole culture was extracted with 27 L ethyl acetate to yield 5 g of dry material. The extract was fractionated by a reversed-phase (C_18_) chromatographic column (20, 40, 60, 80, and 100% MeOH in water). The major metabolite eluted in the 60 and 80% MeOH fractions. These fractions were subjected to further purification by HPLC (Kromasil 100-5-C_18_ 250 × 10 mm, flow rate 2 mL/min, UV 360 nm detection, with 60% aqueous MeOH, 0.1% formic acid) to isolate the major compound. The major compound eluted at 35 min to yield 15 mg as yellow soild. The structure of the major metabolite of the strain PrRe2I22 was identified by NMR spectroscopic analysis including ^1^H, ^13^C, COSY (correlation spectroscopy), HSQC (heteronuclear single quantum correlation), and HMBC (heteronuclear multiple bond correlation) experiments as well as literature comparison (Noguchi et al., 1988). The major metabolite of Group 2 was compared with that of Group 1 because their spectroscopic and chromatographic properties were identical. The major compound produced by Group 3 was identified by the UV and MS data acquired by LC/MS analysis and ^1^H and ^13^C NNR spectra.

### *In vitro* antifungal assay of fungichromin produced by *Streptomyces* spp. strains

Colonies of the fungal strains were inoculated onto the center of 90 mm petri dishes containing YEME agar and incubated at 28°C. After 4 days, four 6 mm diameter sterile paper disks were placed on each inoculated agar petri dish. Sterile paper disks were imbued with three concentrations (10, 20, 50 μL) of fungichromin (10 mg/mL) dissolved in DMSO, and for the negative control 10 μL DMSO was used. Every petri dish was monitored for 28 days.

### Detection of fungichromin directly in an infructescence specimen

An infructescence specimen was dissected into the involucral bracts and florets. Then each part was soaked in MeOH and organic compounds were extracted with MeOH for 3 h. The MeOH extracts were analyzed by LC/MS using the following gradient conditions; 0~20 min: 10~100% CH_3_CN in H_2_O, 20~25 min: 100% CH_3_CN/flow rate: 0.7 mL/min, 0.1% formic acid in CH_3_CN and H_2_O. A diode array UV detector scanned from 200 to 700 nm. The mass spectrometric analyses were performed on both positive and negative ion modes. Matrix-assisted laser desorption/ionization (MALDI) MS was also performed against pure fungichromin and the infructescence specimen. For MALDI MS of pure fungichromin, 0.2 mg/mL fungichromin in MeOH (0.5 μL) was first spotted and dried on the 384-well MALDI target plate (ASTA Inc., Suwon, Korea) followed by 0.5 μL of the matrix solution. The matrix solution was 10 mg/mL 2, 5-dihydroxybenzoic acid (DHB) in acetonitrile/water (50:50 *v/v*). For direct analysis of infructescence specimen, dissected florets were first attached onto the MALDI target plate by using a conductive double-sided tape and gently dried under moderate vacuum (~50 Torr). After drying, the DHB matrix solution was sprayed on a floret surface by using an airbrush. MALDI mass spectra were recorded in the positive reflectron mode by using an ABI 4800 Plus MALDI-TOF/TOF analyzer (Applied Biosystems, Foster City, CA, USA).

## Results

### Isolations of bacterial strains

A total of 83 putative actinomycetes were obtained. All of these isolates could be classified in one of three morphological groups. Isolates residing in *Streptomyces* sp. Group 1 had soft black colonies, produced gray spores and a yellow substrate pigment was observed in surrounding growth medium. Members belonging to *Streptomyces* sp. Group 2 produced yellow translucent colonies with gray spores. A red substrate pigment was produced by all isolates in this group. *Streptomyces* sp. Group 3 had white colonies and produced light gray spores while a red substrate pigment was observed. From the total number of isolates, 20 representing the different morphological groups were selected (Table 1) for further detailed identification.

Isolates of *Streptomyces* sp. Group 1 were from *P*. *repens* in Pringle Bay and Stellenbosch, and *P*. *neriifolia* in Franschhoek. *Streptomyces* sp. Group 2 isolates were from both hosts at all locations sampled, while *Streptomyces* sp. Group 3 isolates were from *P*. *neriifolia* in Pringle Bay and *P*. *repens* in Pringle Bay and Stellenbosch (Table 1).

### DNA sequencing and phylogenetic analysis

**Figure 2:**
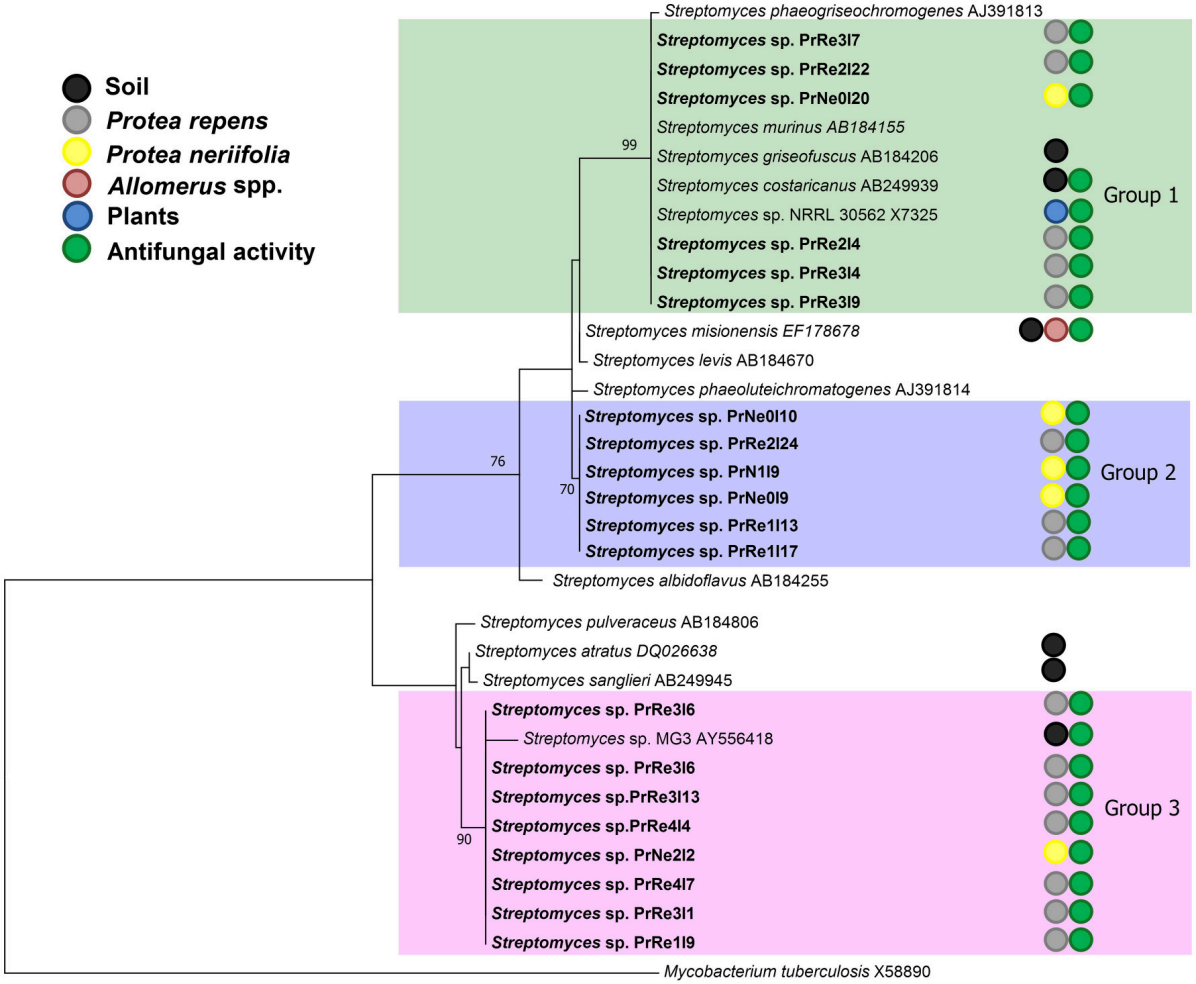
**A ML tree exhibiting the phylogenetic relationships based on 16S rRNA gene sequence of *Streptomyces* species obtained during the present study**. Isolate numbers from this study are printed in bold type. Groups of isolates are indicated by shaded areas.

The aligned 16S rRNA data set consisting of sequences obtained in the present study and those of type strains from RDP database consisted of approximately 650 characters from the species-specific α-region of the 16S rRNA gene sequence (Kataoka et al., 1997). Maximum likelihood analysis confirmed that isolates having a similar morphology also grouped together based on 16S rRNA sequences (Figure 2). Furthermore, all the isolates collected in this study grouped among species of *Streptomyces*.

**Figure 3:**
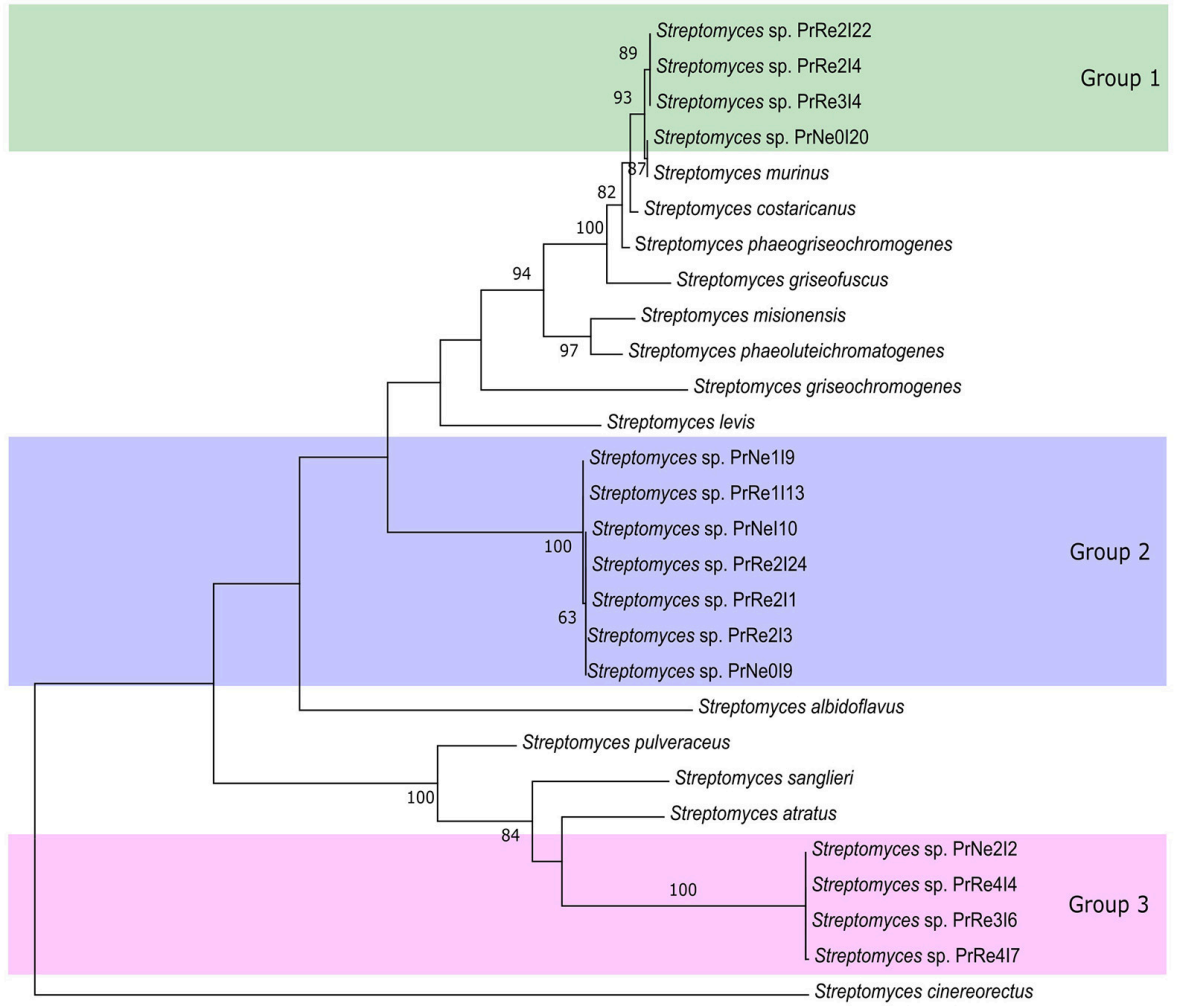
**Maximum likelihood tree showing phylogenetic placement of our isolates based on concatenated 16S rRNA-*trpB*-*rpoB*-*gyrB* alignments**. Only bootstrap values above 65% are indicated. Shaded areas indicate groups of isolates.

According to the 16S rRNA maximum likelihood phylogeny, isolates belonging to *Streptomyces* sp. Group 1 formed a well-supported, distinct clade with the type strains of *S. griseofuscus, S. murinus, S. phaegriseochromogenes, S. costaricanus*, and an undescribed *Streptomyces* sp. NRRL 30562 from snakevine in Australia (Castillo et al., 2002, 2006, Figure 2). The ML phylogenetic tree based on the four combined genes was able to separate our isolates in this group into two clades; one which included isolates from *P. repens* (PrRe2I4, PrRe2I22, PrRe3I4) with 90% bootstrap support, while PrNe0I20 from *P. neriifolia* formed a sister clade and grouped with the type strain of *S. murinus* with 77% bootstrap support (Figure 3). These two clades formed a larger group which was separated (93% bootstrap support) from the *S. costaricanus, S. phaeogriseochromogenes*, and *S. griseofuscus* (Figure 3). Isolates from this group could belong to *S. murinus* or the proposed species *S. padanus* (Xiong et al., 2012).

In the 16S rRNA phylogenetic tree, isolates in *Streptomyces* sp. Group 2 formed its own clade which did not include any type strain sequences (Figure 2). The closest type strains were those from *S. misionensis, S. levis*, and *S. phaeoluteichromatogenes* (Figure 2). In the MLSA phylogenetic tree, these isolates also clustered separately with considerable distance to all type strains and with 100% bootstrap support and thus most likely represents an undescribed species. Interestingly, this clade formed a subgroup containing isolates from both *P. repens* and *P. neriifolia*.

Members of *Streptomyces* sp. Group 3 formed a distinct cluster with 93% bootstrap support with an unidentified strain (MG3) from soil in Germany (Hoster et al., 2005). According to our MLSA, these isolates remained in a distinct cluster, with 100% bootstrap support and were separated from the type strains of *S. atratus* and *S. sanglieri* (Figure 3).

### *In vitro* antifungal assays

**Figure 4:**
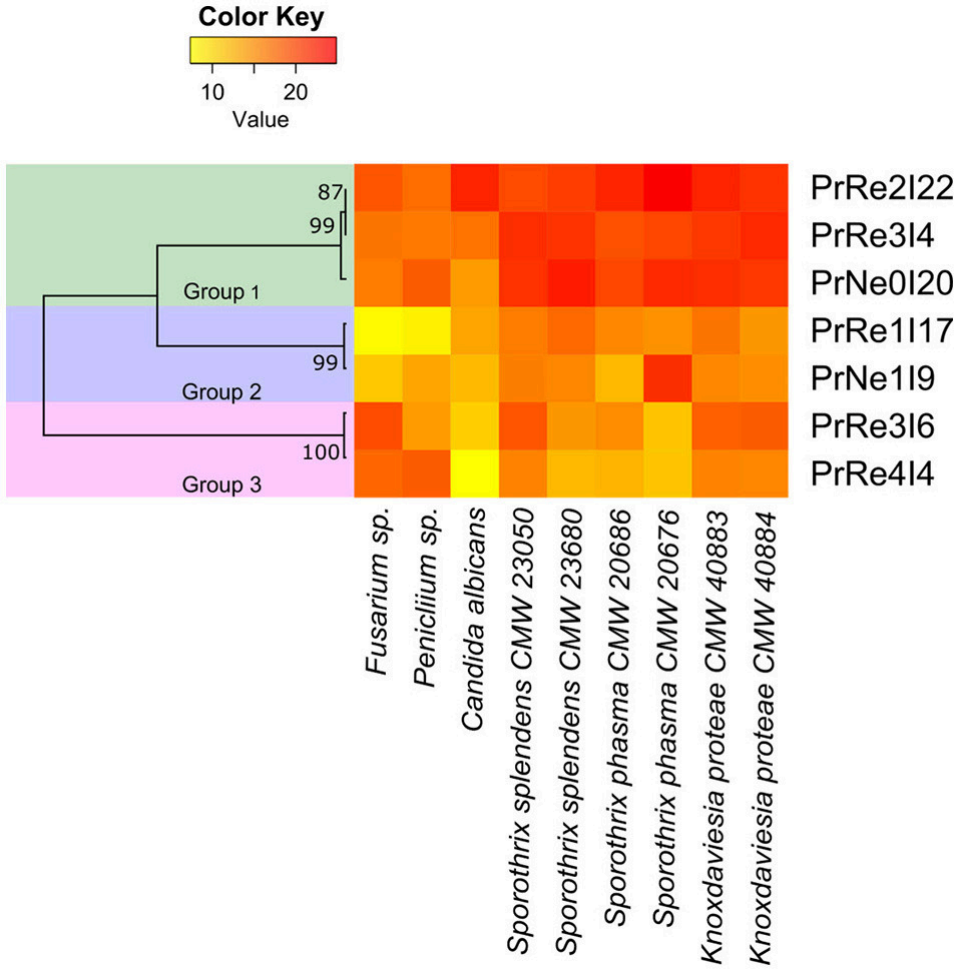
**Heatmap illustrating *in vitro* bioassay results**. Values represented from 0 to 22.8 on a continuous scale from yellow to red. Phylogenetic relationships between bacterial isolates are presented to the left of the heatmap.

Seven bacterial isolates were included in the assays and these represented the three different groups identified from *Protea* infructescences (Table 2, Figure 4). Isolates residing in *Streptomyces* Group 1 generally had strong inhibitory activities against all tested fungi and the antifungal activity against *Sporothrix* spp. and *K. proteae* was generally higher than against saprophytic test fungi (Table 2, Figure 4).

Isolates belonging to *Streptomyces* Group 2 was able to inhibit the growth of all tested fungi, although this was generally limited and much lower than observed for members of Group 1 and only limited inhibition of the two saprophyte test fungi was recorded (Table 2, Figure 4). *Streptomyces* sp. Group 3 had noticeable inhibition against *Penicillium* and *Fusarium* isolates, similar to the inhibition observed for Group 1. Interestingly, isolates from this group had lower levels of inhibition against the *Sporothrix* spp. tested fungi, while *K. proteae* was generally inhibited to levels similar to *Penicillium* and *Fusarium*. The isolates from Group 3 also had much lower inhibitory activity against *C. albicans*.

### Identification of major secondary metabolites produced by *Streptomyces* spp. strains and antifungal activity

**Figure 5:**
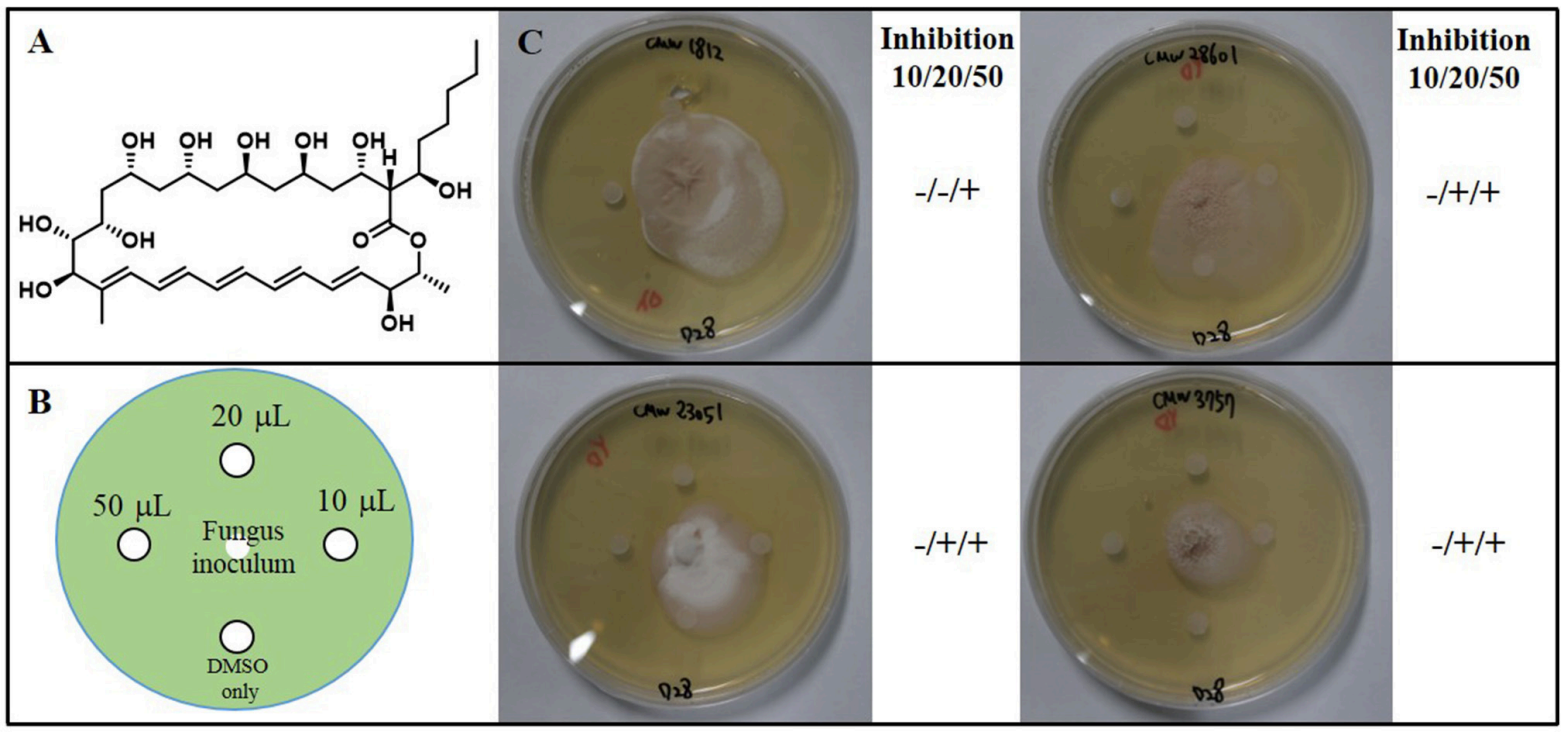
**(A)** The chemical structure of fungichromin. **(B)** Petri plate antifungal activity assay, showing the placement of three concentrations (10 μL, 20 μL, 50 μL) of fungichromin (10 mg/mL) dissolved in DMSO, the control (DMSO only, 10 μL), and the placement of the fungal inoculum. **(C)** Representative image examples of fungichromin activity against *Sporothrix africana* CMW 1812, *S. variecibatus* CMW 23051, *S. protea-sedis* CMW 28601, *Knoxdaviesia proteae* CMW 3757, 28 days after inoculation.

To identify secondary metabolites from the *Streptomyces* spp. strains responsible for the growth inhibition of fungi, we cultivated Groups 1 to 3 *Streptomyces* spp. strains in liquid culture medium. Based on the LC/MS (liquid chromatography and mass spectrometry) chemical profiling of the EtOAc extracts of the cultures, a common major secondary metabolite in Group 1 and 2 strains was distinctively detected. This compound displayed a typical UV spectral feature (λ_max_ at 325, 340, and 362 nm), indicating the existence of five conjugated double bonds, and the low resolution molecular ion [M+Na]^+^ at *m*/*z* 693. Comparison of the UV spectrum of the compound with our house-built UV library interlinked with the LC/MS software clearly indicated that this metabolite was similar to pentamycin, which bears a pentaene moiety. For definite structure identification, a large culture (18 L) of the *Streptomyces* sp. strain (PrRe2I22) was prepared. Subsequently, we analyzed ^1^H (Supplementary Figure S1), ^13^C (Supplementary Figure S2), and two-dimensional NMR spectra of the compound including ^1^H-^1^H correlation (COSY; Supplementary Figure S3), heteronuclear single quantum coherence (HSQC; Supplementary Figure S4), and heteronuclear multiple bond correlation (HMBC; Supplementary Figure S5). The spectroscopic analysis and the comparison of the spectroscopic data with the literature (Noguchi et al., 1988) unequivocally identified the compound of Groups 1 and 2 as fungichromin, which is also called pentamycin, a polyene polyketide secondary metabolite as we expected (Figure 5). Therefore, the major secondary metabolite of Groups 1 and 2 *Streptomyces* strains was elucidated as fungichromin. We confirmed that fungichromin is responsible for the antifungal activity observed using an antifungal assay (Figure 5). Petri dishes were observed daily for 28 days (Figure 5). Fungichromin inhibited the growth of the fungi in a dose-dependent manner (Figure 5).

**Figure 6:**
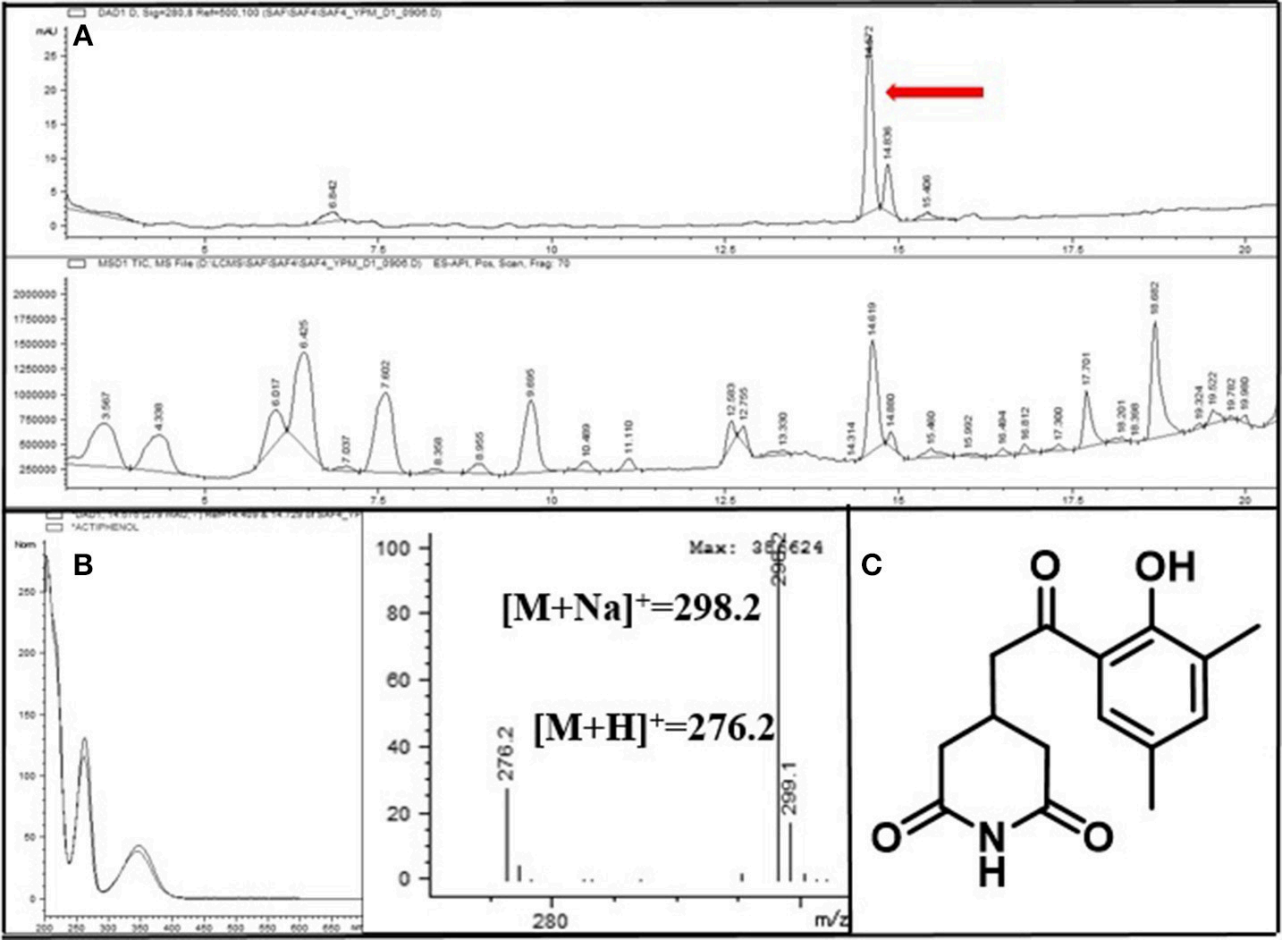
**(A)** LC/MS chemical analysis of the liquid culture of the *Streptomyces* strain PrRe3I6. UV chromatogram at 280 nm (upper), and mass chromatogram (lower). **(B)** UV and mass spectra of the major secondary metabolite, actiphenol. **(C)** The chemical structure of actiphenol.

A common major secondary metabolite in Group 3 strains was also detected. This compound showed a distinct UV spectral feature (λ_max_ at 220, 260, and 350 nm) and the low resolution molecular ion [M+H]^+^ at *m*/*z* 276. Compared with our house-built UV library, the molecular ion detected in MS, and ^1^H and ^13^C NMR spectra (Supplementary Figures S6, S7), this metabolite was identified as actiphenol (Highet and Prelog, 1959; Sun et al., 2014), which belongs to the cycloheximide class (Figure 6).

### Detection of fungichromin in plant material

**Figure 7:**
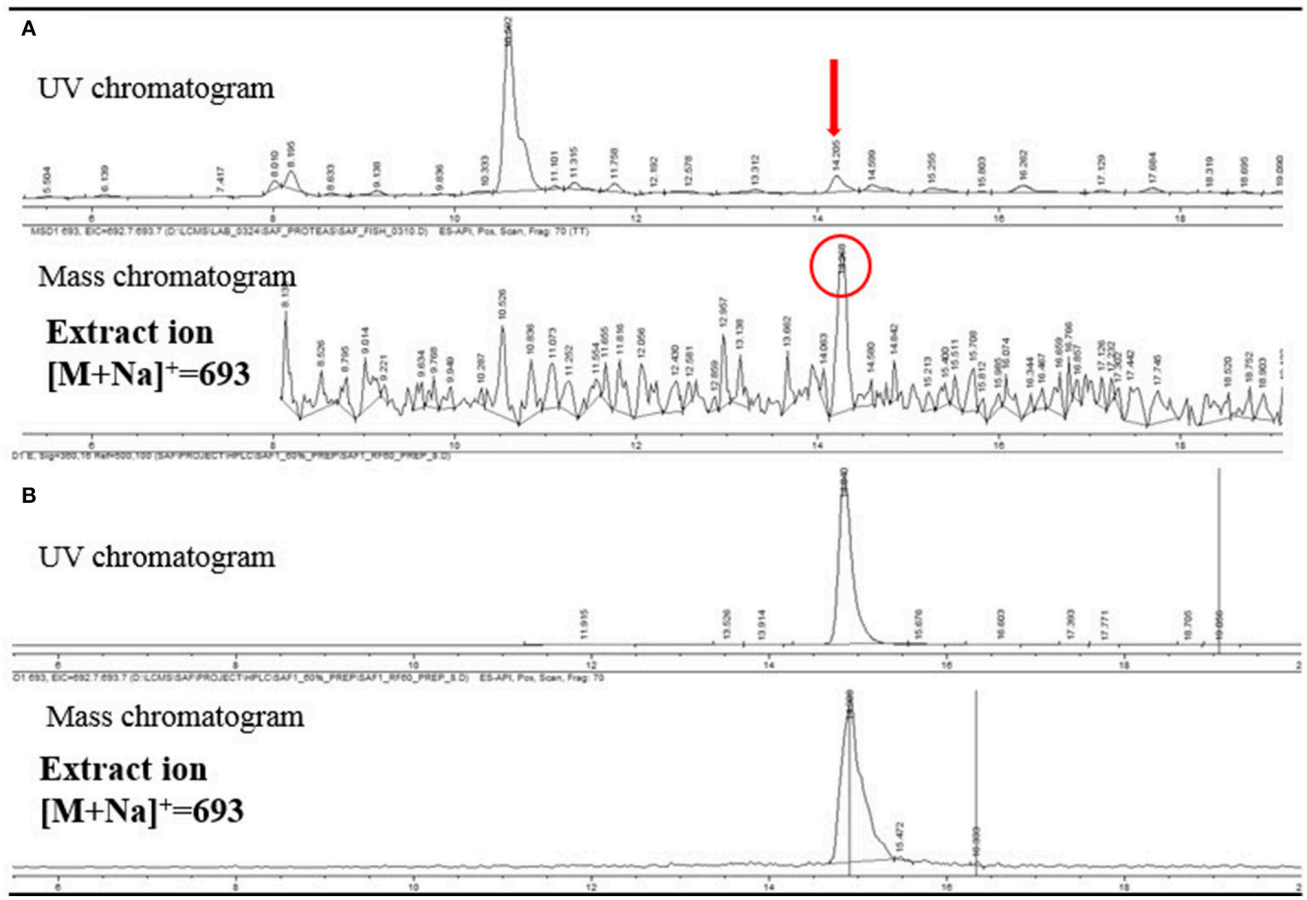
**(A)** LC/MS analysis of the extract of a *Protea* infructescence specimen. UV chromatogram (upper) and mass chromatogram (lower). **(B)** LC/MS analysis of pure fungichromin (UV detection at 360 nm).

**Figure 8:**
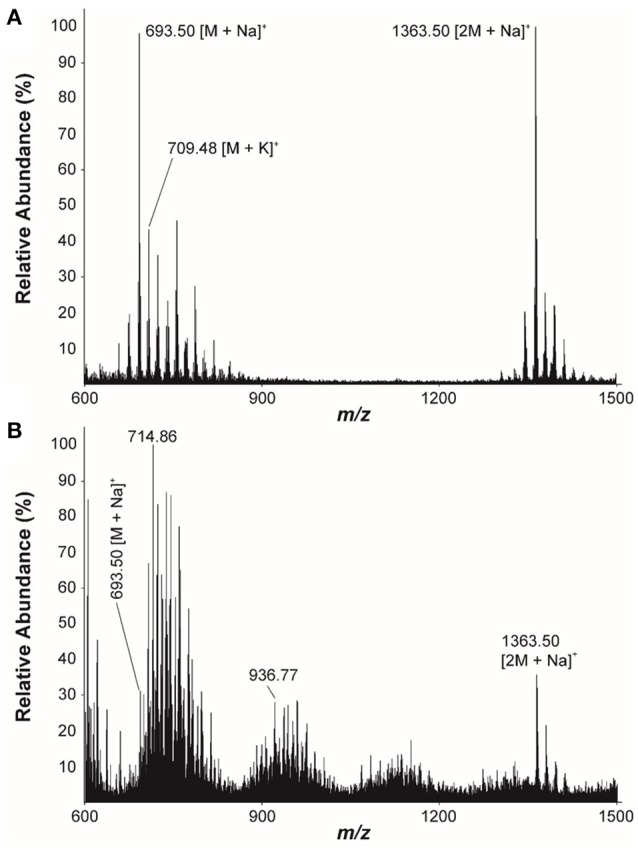
**Positive ion-mode MALDI mass spectra of (A) The pure fungichromin and (B) The infructescence specimen (floret part)**.

The LC/MS analysis of the MeOH extraction of the infructescence specimen clearly detected fungichromin in the involucral bracts and florets. The exact ion [M+Na]^+^ at *m*/*z* 693 was observed at the identical retention time to that of pure fungichromin (Figure 7). We also attempted to detect fungichromin directly from the infructescence specimen by MALDI MS. Figure 8 shows MALDI mass spectra of pure fungichromin and the floret part of the infructescence specimen. For the pure fungichromin, sodium and potassium adduct ions of fungichromin ([M + Na]^+^ and [M + K]^+^) were clearly detected at *m/z* 693.50 and 709.48, respectively (Figure 8A). In addition to these ions, another intense signal was observed at *m/z* 1363.50 and this signal was found to be sodium adduct ions of fungichromin dimer ([2M + Na]^+^) by MALDI tandem MS (MS/MS). After confirming that fungichromin is readily detected by MALDI MS, we performed direct analysis of the infructescence specimen by MALDI MS. Figure 8B shows a chemical fingerprint obtained directly from this specimen. From the floret part of the infructescence specimen, both sodium adduct ions of fungichromin and its dimer were detected by MALDI MS. Although the signal corresponding to [M + Na]^+^ at *m/z* 693.50 was crowded by other metabolite ion signals, the signal corresponding to [2M + Na]^+^ at *m/z* 1363.50 was distinctly visible (Figure 8B).

## Discussion

This study represents the first discovery of antibiotic producing actinomycete bacteria in the infructescences of *Protea* spp. In all, three main groups of actinomycetes were identified and they were all species of *Streptomyces*. Bioassays showed that some of these bacteria were antagonistic to saprotrophic fungi found on the outside of *Protea* infructescences. Results of this study suggest that the presence of actinomycetes that produce antifungal compounds might contribute to the absence of saprotrophic fungi in the nutrient rich, moist and warm environment found in *Protea* infructescences (Roets et al., 2012).

Isolates from *Streptomyces* spp. Group 1 had identical 16S rRNA sequences to *Streptomyces* sp. NRRL 30562 (Castillo et al., 2002), an endophyte from *Kennedia nigriscans* and identified as *S. padanus*, a species that has not yet been validly described. The type strain most closely resembling our isolates was *S. murinus* (Frommer, 1959). Strains identified as *S. padanus*, isolated from soil (Xiong et al., 2012) has also been reported from mountain laurel (*Streptomyces* sp. AOK-30) (Ericaceae) (Nishimura et al., 2002), which could be reinfected to become more resistant to fungal pathogens (Nishimura et al., 2002). *Streptomyces padanus* generally produces the antibiotics actinomycin (Kurosawa et al., 2006) and fungichromin (Shih et al., 2003; Xiong et al., 2012). Fungichromin was also identified as the active compound associated with this group of isolates. Production of this compound under natural conditions was also demonstrated as it could be detected in the infructescence material.

Our MLSA showed that *Streptomyces* sp. Group 2 is an undescribed species and most closely resemble the type strains of *S. phaeoluteichromatogenes, S. misionensis*, and *S. levis*. The origin of *S. phaeoluteichromatogenes* and *S. levis* is not known, but *S. misionensis* was originally isolated from soil in Argentina. Strains identified as *S. misionensis* have been used as biocontrol agents against *Fusarium* pathogens of *Lilium* spp. (Chung et al., 2011). *Streptomyces misionensis* has also been detected on ants (*Allomerus* spp.) and was found to antagonize fungal pathogens of the ant colonies (Seipke et al., 2012).

*Streptomyces* sp. Group 3 was shown to be an undescribed species, most similar to *S. atratus* and *S. sanglieri*, both species of which have been reported from soil (Manfio et al., 2003). *Streptomyces sanglieri* produces the antibiotic lactonamycin Z that has antibacterial and antitumor activity (Zhang et al., 2008). *Streptomyces* sp. MG3, the closest matching sequence from Genbank, was isolated from soil in Germany and had strong antifungal activity due to the production of a chitinase (Hoster et al., 2005).

All three groups of isolates were found on both *P. repens* and *P. neriifolia* infructescences, and it is clear that actinomycetes are commonly present in the infructescences of *Protea* plants. Our results are concordant with other studies that detected antibiotic producing *Streptomyces* spp. in plants, with one from a member of the family Proteaceae. Isolates residing in *Streptomyces* sp. Group 1 are very similar to those found in *K. nigriscans* in Australia, which produce munumbicin A, B, C, and D, active against many pathogenic bacteria and fungi (Castillo et al., 2002, 2006). *Grevillea pteridifolia*, a member of the Proteaceae from Australia, is a host to *Streptomyces* spp. that produce the range of antibiotics known as the kakadumycins (Castillo et al., 2003). *Monstera* sp. harbors *Streptomyces* endophytes producing the coronamycin antibiotics (Ezra et al., 2004). All of these are thought to provide some protection to the host plant (Strobel and Daisy, 2003).

Most of the species related to the isolates obtained in this study originate predominately from soil, where *Streptomyces* spp. most commonly occur (Kampfer, 2006). Members of the mite family Edbakerellidae commonly occur in soil, and several new species of this family have recently been discovered in *Protea* infructescences (Theron et al., 2012). *Streptomyces* spp. have spores that are well-adapted to arthropod dispersal (Ruddick and Williams, 1972; Chater, 2006). Furthermore, it has been shown that *S. griseus* spores adhere to the exoskeletons of mites, effectively facilitating its spread (Goodfellow and Williams, 1983) and it is reasonable to hypothesize that these mites play a role in maintaining *Streptomyces* spp. in *Protea* infructescences.

Streptomycetes are able to digest several complex carbohydrates and nitrogenous wastes including a multitude of nutrients inaccessible to many other organisms (Kaltenpoth, 2009). They also utilize chitin from fungal debris or insect exoskeletons, lignocellulose, and several other plant associated polymers (Crawford, 1978; Kampfer, 2006). Due to the potential of *Streptomyces* to grow in very diverse environments and the ease with which they are dispersed by arthropods (Ruddick and Williams, 1972; Goodfellow and Williams, 1983), they could potentially play an important role in the interaction between insects, mites, fungi, and *Protea* spp.

All three groups of *Streptomyces* spp. isolated in this study had noticeable antifungal activity. Members from *Streptomyces* spp. Group 1 had the strongest antifungal activity against all tested fungi. Fungichromin, a polyene antifungal compound, is known to inhibit a broad spectrum of fungi (Dixon and Walsh, 1996). *Streptomyces* spp. Groups 2, of which the major secondary metabolite is also fungichromin, had stronger inhibitory activity against the ophiostomatoid fungi than against the non-ophiostomatoid saprophytes, possibly due to their slower growth and an optimal level of fungichromin production. Members of *Streptomyces* spp. Group 3 produced actiphenol, a compound belonging to the antifungal cycloheximide class (Highet and Prelog, 1959). This group allowed for better growth of *Sporothrix* spp. and *C. albicans*, known to be tolerant of cycloheximide (Yamada-Okabe and Yamada-Okabe, 2002; Roets et al., 2008). No possible benefit to *K. proteae* could be deduced from *in vitro* bioassay results, but it is clear that common saprotrophic fungi are sensitive to *Streptomyces* spp. from *Protea* plants.

There are two potential benefits provided to the plant by actinomycete bacteria. *Protea* infructescences provide an enclosed environment with the ideal requirements for fungal growth. They are, therefore, at some risk from invasion by degrading saprophytes and pathogens. Furthermore, once seeds have spread and come into contact with soil, they will come into contact with more potentially harmful microbes. *Streptomyces* spp. have the ability to provide continued protection as has been shown in maize where the levels of seed pathogenic fungi have been reduced through treatment with a mixture of *Streptomyces* strains (Bressan, 2003). Thus, *Streptomyces* spp. most likely provide various advantages to *Protea* infructescences and these would have provided a positive force to maintain the relationship between them.

Future studies should determine the mechanism of inoculation of *Streptomyces* spp. in *Protea* spp., including their ability to be spread and grazed upon by arthropods inhabiting *Protea* infructescences. Possible mutualistic interactions need to be investigated, which can be achieved by wider sampling across geographic regions and over time, and should include the effect of more variables such as temperature, rainfall, soil type, and associated animal diversity. Functional metagenomics is an emerging field of study that may also be applied to this niche. Knowledge of the fungal and bacterial secondary metabolite gene clusters present in this niche may provide very important answers toward understanding the complex interactions between *Protea* spp. and the diversity of microorganisms and animals associated with them.

## Author contributions

MW, ZdB, BS, ZH, and SV conceptualized the study. ZH did isolations and assays in the laboratory. ZH, ZdB, and SV did phylogenetic and assay data analyses. DO designed chemical analysis and interpreted chemical data. KM performed LC/MS and NMR analysis and identified antifungal compounds. MB purified and identified actiphenol by NMR spectroscopic data. SC contributed to mass spectrometric analysis. ZH, SV, KM, DO, ZdB, SC, BS, and MW wrote and edited the text. ZH and KM contributed equally to this work.

## Funding

We acknowledge financial support from the Department of Science and Technology (DST)-National Research Foundation (NRF) Centre of Excellence in Tree Health Biotechnology (CTHB), the National Research Foundation (NRF), and the University of Pretoria, South Africa. This work was also supported by a National Research Foundation of Korea Grant funded by the Korean Government (Ministry of ICT and Future Planning; No. 2014R1A2A1A11053477).

### Conflict of interest statement

The authors declare that the research was conducted in the absence of any commercial or financial relationships that could be construed as a potential conflict of interest.
