# Supplementary material for: Antifungal Streptomyces spp. Associated with the Infructescences of Protea spp. in South Africa
Source: Front Microbiol. 2016 Nov 2;7:1657. doi: 10.3389/fmicb.2016.01657 (PMC5090004; doi:10.3389/fmicb.2016.01657)
Supplement: Supplementary file 1 [file DataSheet1.pdf]

frontiers

Supplementary Material

Antifungal *Streptomyces* spp. associated with the infructescences of two *Protea* spp. in South Africa

Zander R Human[1,†], Kyuho Moon[2,†], Munhyung Bae[2], Z. Wilhelm de Beer[1], Sangwon Cha[3], Michael J. Wingfield[1], Bernard Slippers[4], Dong-Chan Oh[2,*], and Stephanus N. Venter[1,*]

[1] Department of Microbiology and Plant Pathology, Forestry and Agriculture Biotechnology Institute (FABI), University of Pretoria, Pretoria, South Africa

[2]Natural Products Research Institute, College of Pharmacy, Seoul National University, Seoul, Republic of Korea

[3]Department of Chemistry, Hankuk University of Foreign Studies, Yongin, Republic of Korea

[4] Department of Genetics, Forestry and Agriculture Biotechnology Institute (FABI), University of Pretoria, Pretoria, South Africa

[†]These authors contributed equally to this work.

*** Correspondence:**
Dong-Chan Oh
dongchanoh@snu.ac.kr

Stephanus N. Venter
Fanus.Venter@up.ac.za

1 Supplementary Figures and Tables

1.1 Supplementary Figures

Supplementary Figure S1. ^{1}H NMR spectrum (600 MHz) of fungichromin in CD_3OD.

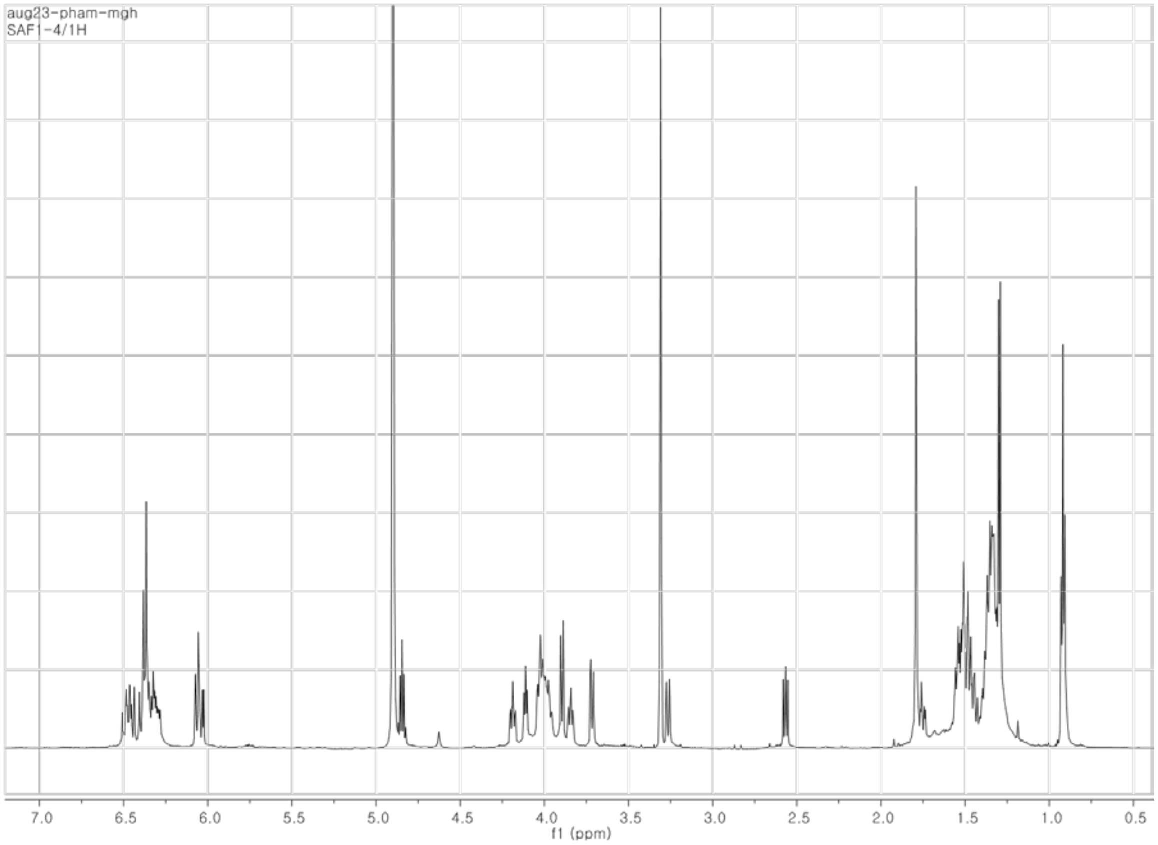

Supplementary Figure S2. ^{13}C NMR spectrum (150 MHz) of fungichromin in CD_3OD.

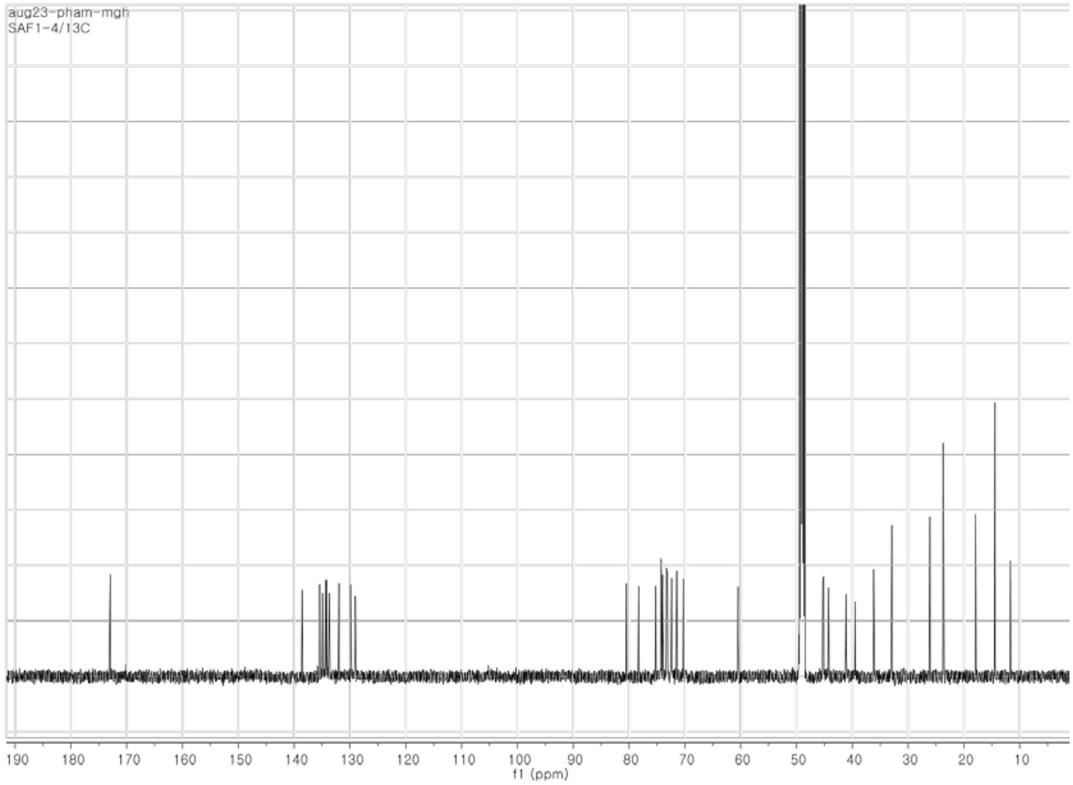

Supplementary Figure S3. COSY spectrum (600 MHz) of fungichromin in CD_3OD.

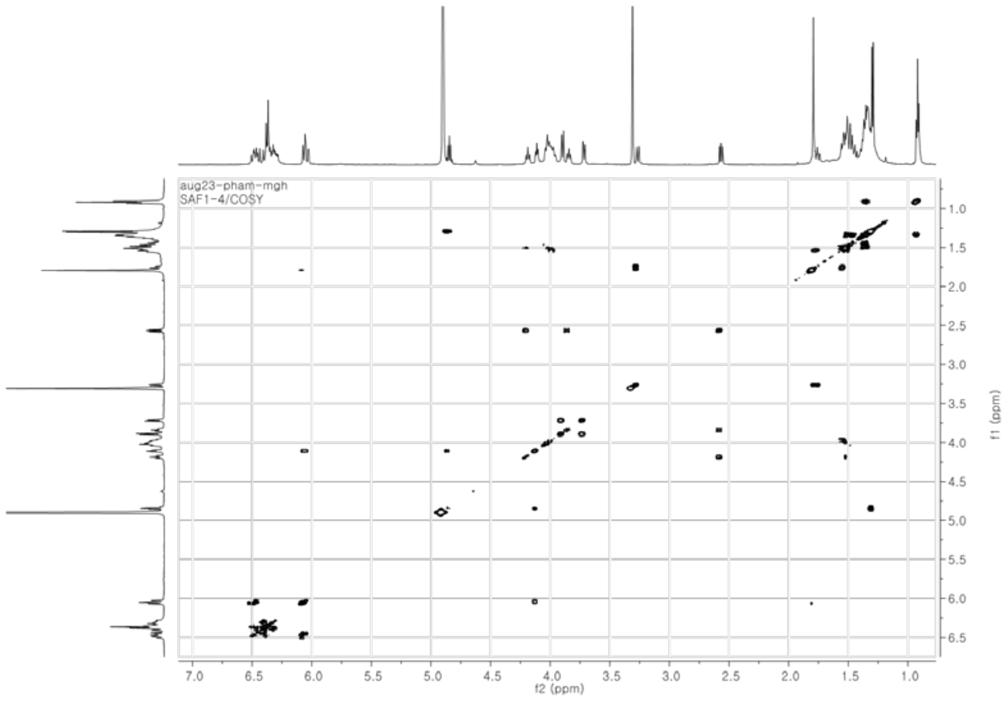

Supplementary Figure S4. HSQC spectrum (600 MHz) of fungichromin in CD_3OD.

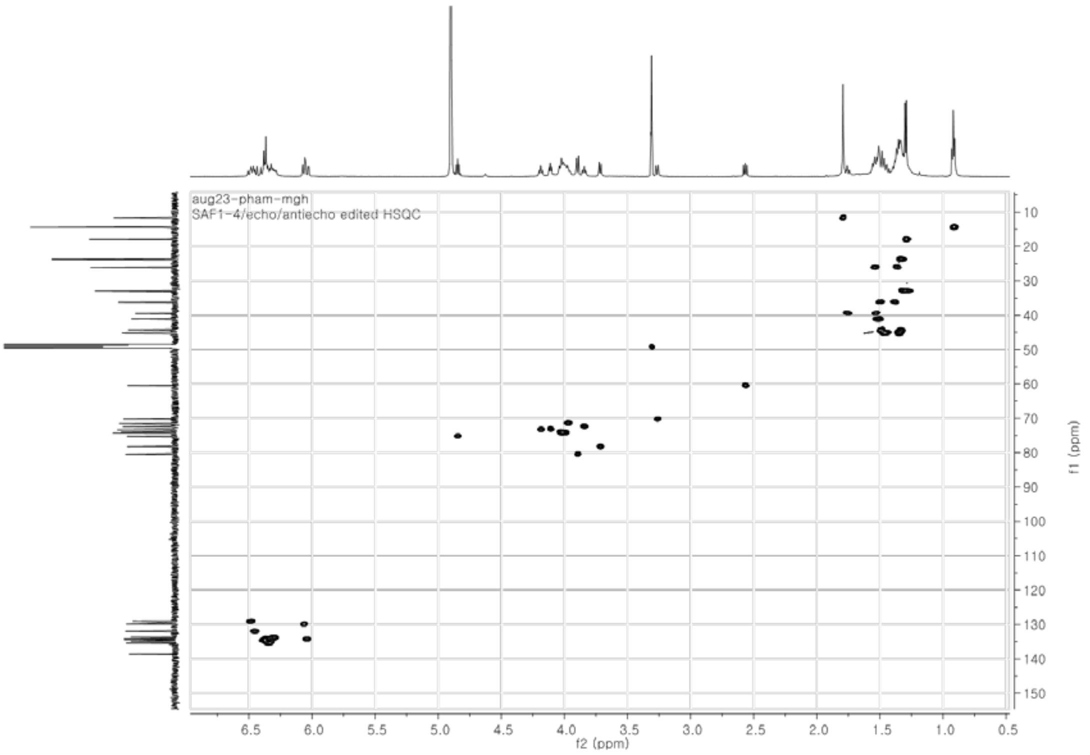

Supplementary Figure S5. HMBC spectrum (600 MHz) of fungichromin in CD_3OD.

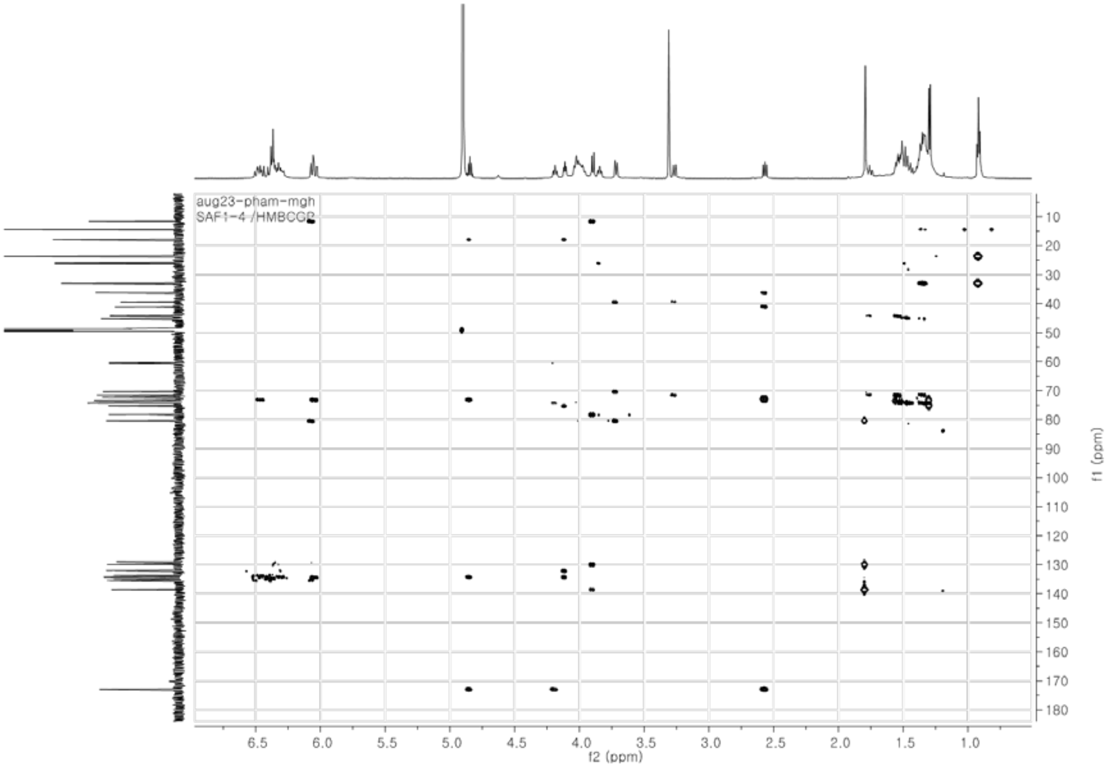

Supplementary Figure S6. ^{1}H NMR spectrum (600 MHz) of actiphenol in DMSO-d_6.

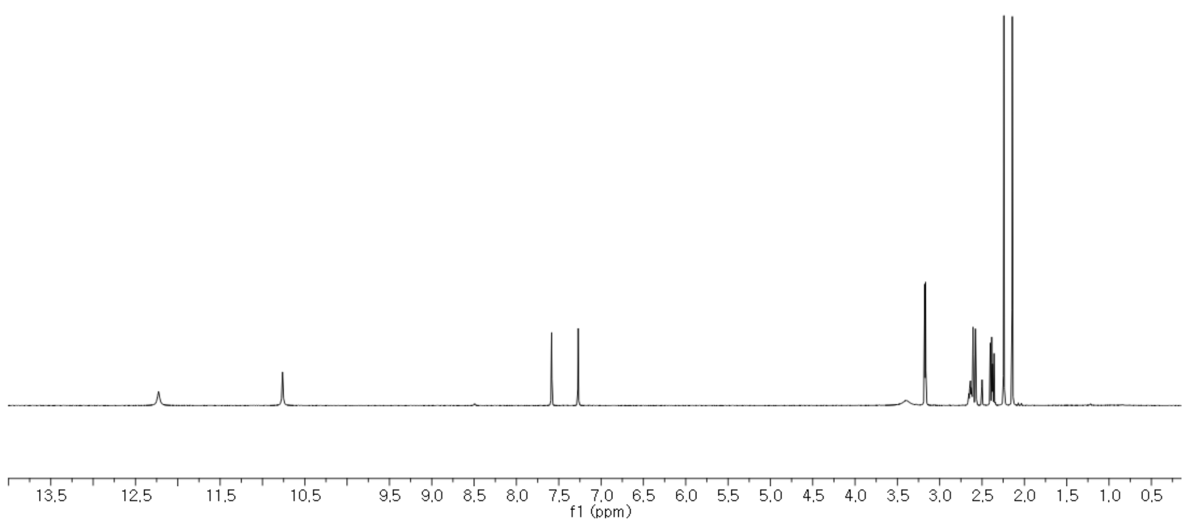

Supplementary Figure S7. ^{13}C NMR spectrum (150 MHz) of actiphenol in DMSO-d_6.

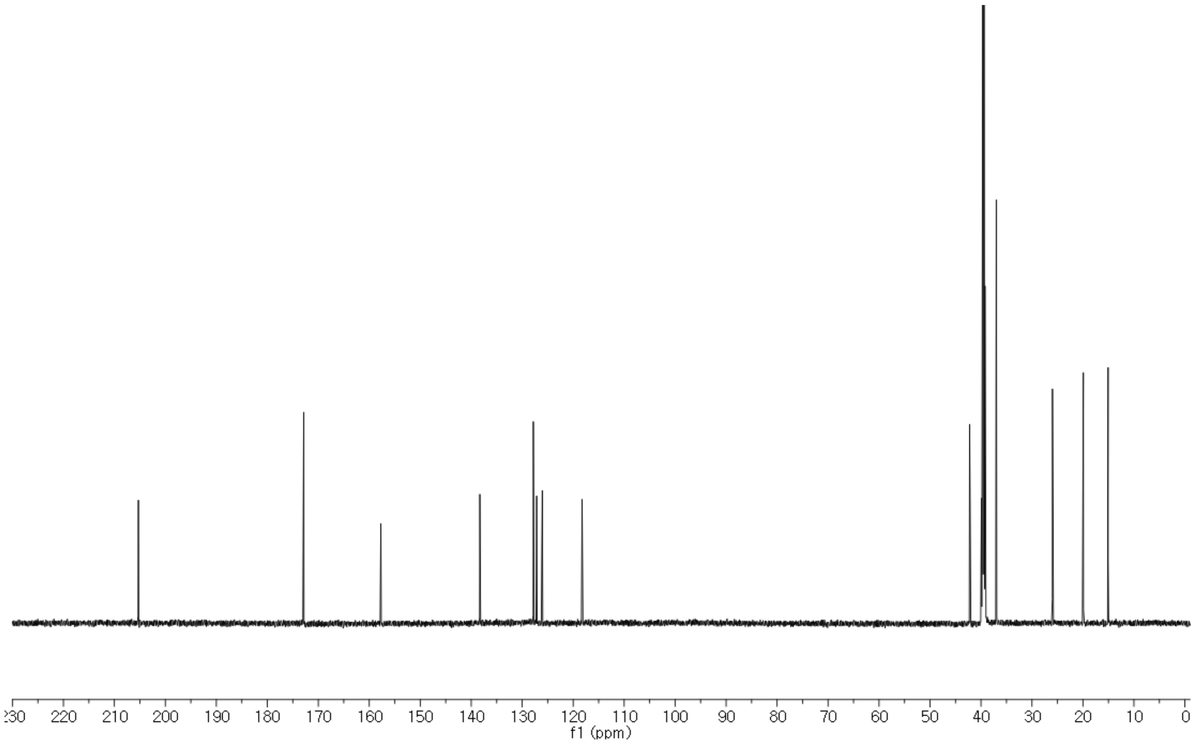

1.2 Supplementary Tables

Supplementary Table S1. *Streptomyces* isolates from this study and others used in MLSA and their Genbank Accession numbers

Isolate name	Strain number	*gyrB*	*trpB*	*rpoB*
S. murinus	NRRL B-2286	KX685590	KX685586	KX685588
S. costaricanus	NRRL B-16897	KX685591	KX685587	KX685589
S. phaeogriseichromatogenes	NRRL 2834	KX685592	KX685593	KX685594
S. griseofuscus	NRRL B-5429	JOFU01000001	JOFU01000006	JOFU01000024
S. phaeoluteichromatogenes	NRRL B-5799	HG423666	HG423654	HG423678
S. misionensis	CBS 885.69	HG423667	HG423655	HG423679
S. griseochromogenes	NRRL B-12423	CP016279	CP016279	CP016279
S. levis	NRRL B-16370	HG423670	HG423660	HG423682
S. albidoflavus	Rong *et al.* (2008)	FJ406417	FJ406450	FJ406439
S. pulveraceus	Guo *et al.* (2008)	HQ823601	EF055175	EF055120
S. sanglieri	Rong *et al.* (2010)	KU049714	KU049720	KU049718
S. atratus	Rong *et al.* (2010)	EF661728	EF661791	EF661770
S. cinereorectus	Rong *et al.* (2008)	EF661732	EF661795	EF661774
Streptomyces sp.	PrRe2I22	KX685530	KX685560	KX685545
Streptomyces sp.	PrRe2I4	KX685531	KX685561	KX685546
Streptomyces sp.	PrRe3I4	KX685532	KX685562	KX685547
Streptomyces sp.	PrNe0I20	KX685529	KX685559	KX685544
Streptomyces sp.	PrNe1I9	KX685522	KX685552	KX685537
Streptomyces sp.	PrRe1I13	KX685528	KX685558	KX685543
Streptomyces sp.	PrNeI10	KX685523	KX685553	KX685538
Streptomyces sp.	PrRe2I24	KX685526	KX685556	KX685541
Streptomyces sp.	PrRe2I1	KX685525	KX685555	KX685540
Streptomyces sp.	PrRe2I3	KX685527	KX685557	KX685542
Streptomyces sp.	PrNe0I9	KX685524	KX685554	KX685539
Streptomyces sp.	PrNe2I2	KX685533	KX685563	KX685548
Streptomyces sp.	PrRe4I4	KX685534	KX685564	KX685549
Streptomyces sp.	PrRe3I6	KX685535	KX685565	KX685550
Streptomyces sp.	PrRe4I7	KX685536	KX685566	KX685551
